# Prevalence of SARS-CoV-2 infection among urban cleaning and solid waste management workers during transmission of the Omicron variant in Brazil

**DOI:** 10.4178/epih.e2023025

**Published:** 2023-02-16

**Authors:** Paulo Ricardo Martins-Filho, Joyce Thayane da Conceição dos Santos, Márcia Santos Rezende, Fernanda Oliveira de Carvalho, Érica Santos dos Reis, Waneska de Souza Barboza, Taise Ferreira Cavalcante, Cliomar Alves dos Santos, Lucindo José Quintans-Júnior, Renata Grespan, Cristiane Bani Corrêa, Tatiana Rodrigues de Moura, Dulce Marta Schimieguel, Jullyana de Souza Siqueira Quintans, Adriano Antunes de Souza Araújo

**Affiliations:** 1 Federal University of Sergipe, Aracaju, Brazil; 2 Aracaju City Hall, Municipal Health Department, Aracaju, Brazil; 3 Government of Sergipe State, Health Foundation Parreiras Horta, Central Laboratory of Public Health (LACEN/SE), Aracaju, Brazil

**Keywords:** COVID-19, SARS-CoV-2, Waste management, Sanitation

## Abstract

This study estimated the prevalence of severe acute respiratory syndrome coronavirus 2 (SARS-CoV-2) infection in urban cleaning and solid waste management workers during the transmission of the Omicron variant in one of the poorest regions of Brazil (the state of Sergipe). Nasopharyngeal swabs were collected from 494 workers, and the presence of SARS-CoV-2 RNA was tested by quantitative reverse-transcriptase polymerase chain reaction. Data on socio-demographic characteristics, comorbidities, vaccination status, mask use, and use of public transport to commute to the workplace were collected. The prevalence with a 95% confidence interval (CI) was calculated from the proportion of SARS-CoV-2 positive cases among the total number of individuals tested. The prevalence ratio (PR) with a 95% CI was the measure of association used to evaluate the relationship between SARS-CoV-2 infection and the exposure variables. The prevalence of SARS-CoV-2 infection was 22.5% (95% CI, 19.0 to 26.4). Individuals under the age of 40 had a higher prevalence of infection (PR, 1.53; 95% CI, 1.03 to 2.30) as well as those who did not believe in the protective effect of vaccines (PR, 1.78; 95% CI, 1.05 to 2.89). Our results indicate the need for better guidance on preventive measures against coronavirus disease 2019 among urban cleaning and solid waste management workers.

## INTRODUCTION

Urban cleaning and solid waste management workers play a vital role in communities by collecting accumulated solid waste and recycling items from residential areas, commercial centers, industrial companies, and public parks. In Brazil, more than 370,000 people work in this activity and collect 135,000 tons of garbage per day, including domestic and public waste. Most of these workers have low education and earn just over US$300 per month [1].

In the coronavirus disease 2019 (COVID-19) pandemic, waste collectors have been one of the highest occupational risk groups for severe acute respiratory syndrome coronavirus 2 (SARS-CoV-2) infections, along with healthcare professionals [2]. Under experimental conditions, SARS-CoV-2 has been shown to remain viable for up to 72 hours on plastic, stainless steel, copper, and cardboard [3]. COVID-19 patients undergoing home treatment may dispose of infectious household waste, which can pose a risk to workers and the environment [4]. Concerns have been raised regarding the disposal of contaminated face masks as general waste. In addition, waste management is an essential service and has operated continuously during the pandemic, even in areas categorized as high risk for COVID-19.

However, studies evaluating the exposure of urban cleaning and solid waste management workers to COVID-19 are rare. The present study estimated the prevalence of SARS-CoV-2 infection in this population during the transmission of the Omicron variant in one of the poorest regions of Brazil.

## MATERIALS AND METHODS

### Study design and context

This was a cross-sectional study carried out in Aracaju, a city in the state of Sergipe in Northeast Brazil, from February 1, 2022 to February 8, 2022, and included urban cleaning and solid waste management workers who worked in the city. Aracaju is a port city and the state capital of Sergipe, with an area of 182.2 km2 and an estimated population of over 600,000 inhabitants. Furthermore, more than one-third of families are low-income, and the Human Development Index is 0.770.

### Data collection and biological sample

Data and biological materials were collected from urban cleaning and solid waste management workers at the headquarters of the company responsible for waste collection in the city. Testing was voluntary and free of charge and was performed in collaboration with the Municipal Health Department of Aracaju. Workers who took the test provided information on their socio-demographic characteristics, the presence of comorbidities, vaccination status, mask use, and use of public transport to commute to the workplace. There was no restriction regarding age. During the collection period, trained assistants collected nasopharyngeal swabs bilaterally to perform tests using the quantitative reverse-transcriptase polymerase chain reaction (RT-qPCR) technique.

### RNA isolation

The assay was performed according to the manufacturer’s instructions by first extracting a 400-μL aliquot of the specimen into a commercial universal transport medium using the MagMAX Viral/Pathogen Nucleic Acid Isolation Kit and the KingFisher Flex Purification system (Thermo Fisher Scientific, Waltham, MA, USA). In addition, 10 μL of MS2 phage control was added to all specimens and to the negative control, which was used as an internal process control. The nucleic acid was eluted in 50 μL of elution solution.

### Reverse-transcriptase polymerase chain reaction

SARS-CoV-2 was detected with 5 μL of extracted RNA using the TaqPath COVID-19 CE-IVD RT-PCR Kit (Thermo Fisher Scientific), which has both a sensitivity and specificity of 100% (https://www.thermofisher.com/document-connect/document-connect.html?url= https%3A%2F%2Fassets.thermofisher.com% 2FTFS-Assets%2FGSD%2FReference-Materials%2Ftaqpath-ceivdrt-pcr-kit-technical-bulletin.pdf). The assay targeted 3 gene sequences (N2, ORF1ab, and S genes) and MS2, and was performed according to the manufacturer’s instructions (https://assets.thermofisher.com/TFS-Assets/LSG/manuals/MAN0019215_TaqPathCOVID-19_CE-IVD_RT-PCR%20Kit_IFU.pdf). In brief, for each specimen, a master mix was prepared that contained TaqPath 1-Step Multiplex Master Mix (No ROX) COVID-19 real-time PCR assay multiplex, and nuclease-free water. Each run also included a SARS-CoV-2 positive control and a negative control. RT-qPCR analysis was performed on a QuantStudio 5 Real-Time PCR system (Thermo Fisher Scientific).

### Statistical analysis

The outcome of interest was the prevalence of SARS-CoV-2 infection among urban cleaning and solid waste management workers. The point prevalence with a 95% confidence interval (CI) [5] was calculated from the proportion of SARS-CoV-2 positive cases diagnosed through RT-qPCR in the total number of individuals tested. The prevalence ratio (PR) with a 95% CI was the measure of association used to evaluate the relationship between SARSCoV-2 infection and the exposure variables (sex [male and female], age [up to 39 and ≥ 40 years], use of public transport [yes or no] , frequency of use of public transport [1-2, 3-4, ≥ 5 times/wk], complete vaccination schedule [yes or no], and confidence in the protective effect of vaccines [yes, no or no response]). Statistical analyses were performed using the “epitools” package (https://cran.rproject.org/web/packages/epitools/index.html) in R version 3.5.3 (R Foundation for Statistical Computing, Vienna, Austria).

### Ethics statement

This study is part of the EpiSERGIPE Project, which was approved by the Ethics Committee of the Federal University of Sergipe (protocol 33095120.4.0000.5546).

## RESULTS

From a total of 852 urban cleaning and solid waste management workers, 494 were included in the present study, of whom 399 (80.8%) were male and 95 (19.2%) were female. The mean age was 39.1 years (standard deviation: 11.7 years). In total, 431 (87.2%) individuals reported no pre-existing medical conditions, and 57 (11.5%) reported the presence of hypertension or diabetes. Most workers (n= 416, 84.2%) reported using public transport, with 58 (13.9%) using it 1-2 times/wk, 35 (8.4%) 3-4 times/wk, and 310 (74.5%) more than 5 times/wk. Thirteen workers did not report how frequently they used public transportation. Only 4 (0.8%) individuals reported not wearing a face mask.

Regarding vaccination status, 489 (99%) workers reported having received the first dose of COVID-19 vaccine (344 [70.3%] Pfizer-BioNTech; 56 [11.5%] Oxford-AstraZeneca; 45 [9.2%] CoronaVac; 2 [0.4%] Janssen; and 42 [8.6%] without information on the type of vaccine). Among the vaccinated individuals, 145 (29.7%) had a complete vaccination schedule and 344 (70.3%) were partially vaccinated. Among subjects who received the first dose of the Pfizer-BioNTech vaccine, 115 (33.4%) were fully vaccinated at the time of data collection. For the Oxford-AstraZeneca and CoronaVac vaccines, the complete vaccination frequency was 23.2% (13 of 56) and 26.7% (12 of 45), respectively; 2 individuals were immunized with the Janssen vaccine; and in 3 situations, there was no record of the vaccine used. Fifty-six (11.3%) of the total workers included in the study stated that they did not believe in the protective effect of vaccines, 403 (81.6%) responded positively, and 35 (7.1%) did not respond; only 8 (14.3%) of those who responded negatively about vaccine confidence were fully vaccinated against COVID-19.

Of the 494 urban cleaning and solid waste management workers evaluated, 111 (22.5%; 95% CI, 19.0 to 26.4) were positive for the presence of SARS-CoV-2 according to the RT-qPCR test, and all were asymptomatic at the time of collection of data and biological material. Individuals under the age of 40 had a higher prevalence of infection (PR, 1.53; 95% CI, 1.03 to 2.30), as did those who did not believe in the protective effect of vaccines (PR, 1.78; 95% CI, 1.05 to 2.89). There was no difference in the prevalence of SARS-CoV-2 infection based on the frequency of use of public transportation. Of the 111 positive cases of SARS-CoV-2 infection, 80 occurred among unvaccinated or partially vaccinated workers, and 31 among those fully vaccinated. However, no differences were observed in the prevalence of infection according to the type of vaccine used or the workers’ vaccination status (Table 1).

## DISCUSSION

The first case of COVID-19 in Aracaju was identified on March 14, 2020, and as of March 12, 2022, 150,007 cases and 2,524 deaths associated with COVID-19 had been confirmed. The first 10 weeks of 2022 (January 2 to March 12) were characterized by a sudden increase in the number of cases as a result of community transmission of the Omicron variant, and during this third wave of COVID-19, 21,607 cases and 89 deaths were recorded in the city. COVID-19 vaccination coverage for the general population was 76.8% as of March 12, 2022.

The present study showed that one-fourth of urban cleaning and solid waste management workers were positive for SARS-CoV-2 during the period of community transmission of the Omicron variant. These results are alarming and suggest a high vulnerability of these workers to COVID-19. During the first weeks of 2022, a prevalence of approximately 2% of COVID-19 was estimated for the general population in the city of Aracaju [6]. It is important to highlight that workers involved in garbage collection and waste handling are historically exposed to a number of risks related to occupational health, especially in developing countries, where these professionals work outdoors and in direct contact with poorly packaged materials [7-9].

It has been shown that the risk of exposure to SARS-CoV-2 among these workers is greatest during garbage collection, mechanical handling of compactor trucks, and garbage unloading at the disposal site [10]. In addition, issues related to educational level and personal hygiene, the lack of official safety guidelines, and problems with the supply and control of the use of personal protective equipment by companies are factors that can increase the risk of contamination among these individuals. In a study carried out in the city of São Paulo between March 2020 and March 2021, a positive correlation was observed between the number of household waste collection workers with COVID-19 and the incidence of the disease on collection routes, especially in the regions with worse socioeconomic indicators [11]. There is, therefore, a need for collective efforts involving the general population, service providers, and local government to control the spread of COVID-19 arising from the management of waste collection in cities [12].

There is evidence that education and training are key factors in preventing and reducing occupational injuries and illnesses among urban cleaning and solid waste management workers [13,14]. Furthermore, it has been observed that the perceived susceptibility to and severity of an occupational disease are positive predictors of adherence to the use of personal protective equipment [15]. However, in a context where there is a collective need for vaccination against a highly communicable disease such as COVID-19, higher levels of vaccine hesitancy have been observed among individuals with lower educational levels [16]. In our study, although 99% of workers had received the first dose of the vaccine against COVID-19, approximately 11% reported not believing in the protective effect of vaccines and fewer than 30% had completed their vaccination schedule. We found a higher prevalence of SARS-CoV-2 infection among those who lacked confidence in vaccine protection, indicating the need for an effective education program on disease prevention. Despite these findings, further studies should explore the behavioral factors that contribute to the higher prevalence of COVID-19 in this population.

In summary, this study showed a high prevalence of SARS-CoV-2 infection in urban cleaning and solid waste management workers during transmission of the Omicron variant in Northeast Brazil, with higher rates among younger individuals and among those who did not trust the protective effect of vaccines. These findings highlight the importance of COVID-19 monitoring programs among individuals at higher risk of infection, especially when social restriction measures have been relaxed in Brazil. Moreover, our results indicate the need for better guidance on preventive measures against COVID-19 in this population.

## Figures and Tables

**Table 1.** Prevalence of SARS-CoV-2 infection among urban cleaning and solid waste management workers during community transmission of the Omicron variant in Northeast Brazil in 2022

Variables		RT-qPCR positive/total, n (%)	Prevalence ratio (95% CI)	p-value
Sex				
	Male	82/399 (20.6)	1.00 (reference)	
	Female	29/95 (30.5)	1.50 (0.94, 2.30)	0.075
Age (yr)				
	≤39	68/251 (27.1)	1.53 (1.03, 2.30)	0.028
	≥40	43/243 (17.7)	1.00 (reference)	
Use of public transport				
	No	16/78 (20.5)	1.00 (reference)	
	Yes	95/416 (22.8)	1.11 (0.65, 2.03)	0.712
Frequency of use of public transport (times/wk)^1^				
	1-2	15/58 (25.9)	1.00 (reference)	
	3-4	8/35 (22.9)	0.88 (0.33, 2.22)	0.795
	≥5	71/310 (22.9)	0.89 (0.50, 1.67)	0.653
COVID-19 vaccine - first dose^2^				
	Oxford-AstraZeneca	10/56 (17.9)	1.00 (reference)	
	Pfizer-BioNTech	77/344 (22.4)	1.25 (0.65, 2.72)	0.519
	CoronaVac	12/45 (26.7)	1.49 (0.59, 3.86)	0.356
COVID-19 vaccine - second dose^3^				
	Oxford-AstraZeneca	2/13 (15.4)	1.00 (reference)	
	Pfizer-BioNTech	25/115 (21.7)	1.41 (0.35, 12.31)	0.699
	CoronaVac	3/12 (25.0)	1.63 (0.19, 19.46)	0.626
Vaccination schedule				
	Fully vaccinated	31/145 (21.4)	1.00 (reference)	
	Not vaccinated^4^ or partially vaccinated	80/349 (22.9)	1.07 (0.70, 1.68)	0.753
Confidence in the protective effect of vaccines				
	Yes	85/403 (21.2)	1.00 (reference)	
	Preferred not to answer	5/35 (14.3)	0.68 (0.21, 1.64)	0.411
	No	21/56 (37.5)	1.78 (1.05, 2.89)	0.025

SARS-CoV-2, severe acute respiratory syndrome coronavirus 2; RT-qPCR, quantitative reverse-transcriptase polymerase chain reaction; COVID-19, coronavirus disease 2019; CI, confidence interval.

1 Thirteen individuals did not report the frequency of using public transport.

2 Two subjects received the Janssen vaccine, and 42 subjects had no information about the vaccine received as a first dose.

3 Three individuals had no information about the vaccine received.

4 Only 5 individuals were not vaccinated, of whom 1 tested positive for COVID-19.
